# Epidemiology of American Tegumentary Leishmaniasis and* Trypanosoma cruzi* Infection in the Northwestern Argentina

**DOI:** 10.1155/2016/6456031

**Published:** 2016-09-29

**Authors:** Carlos L. Hoyos, Silvana P. Cajal, Marisa Juarez, Jorge D. Marco, Anahí M. Alberti D'Amato, Melina Cayo, Irma Torrejón, Rubén O. Cimino, Patricio Diosque, Alejandro J. Krolewiecki, Julio R. Nasser, José F. Gil

**Affiliations:** ^1^Instituto de Investigaciones en Enfermedades Tropicales, Sede Regional Orán, Universidad Nacional de Salta, 4530 Salta, Argentina; ^2^Instituto de Patología Experimental (IPE-CONICET), Facultad de Ciencias de la Salud, Universidad Nacional de Salta, 4400 Salta, Argentina; ^3^Instituto de Biología de la Altura (InBiAl), Universidad Nacional de Jujuy, Jujuy, Argentina; ^4^Cátedra de Química Biológica, Facultad de Ciencias Naturales, Universidad Nacional de Salta, 4400 Salta, Argentina; ^5^Instituto de Investigaciones en Energía no Convencional (INENCO-CONICET), Universidad Nacional de Salta, 4400 Salta, Argentina

## Abstract

*Background*. Endemic areas of tegumentary leishmaniasis (TL) in Salta, Argentina, present some overlap zones with the geographical distribution of Chagas disease, with mixed infection cases being often detected.* Objectives*. The purpose of this study was to determine the magnitude of* Leishmania* sp. infection and potential associated risk factors, the serologic prevalence of* T. cruzi,* and the presence of* T. cruzi*-*Leishmania* sp. mixed infection in a region of the northwest of Argentina.* Methods*. Cross-sectional studies were conducted to detect TL prevalence and* T. cruzi* seroprevalence. A case-control study was conducted to examine leishmaniasis risk factors.* Results*. Prevalence of TL was 0.17%, seroprevalence of* T. cruzi* infection was 9.73%, and mixed infection proportion—within the leishmaniasic patients group—was 16.67%. The risk factors associated with TL transmission were sex, age, exposure to bites at work, staying outdoors more than 10 hours/day, bathing in the river, and living with people who had lesions or were infected during the study.* Discussion*. The endemic pattern of TL seems to involve exposure of patients to vectors in wild as well as peridomestic environment. Cases of* T. cruzi* infection are apparently due to migration. Therefore, a careful epidemiological surveillance is necessary due to the contraindication of antimonial administration to chagasic patients.

## 1. Introduction

Tegumentary leishmaniasis (TL) is caused by protozoan parasites belonging to the genus* Leishmania*. Clinical manifestations of this disease include single, multiple, disseminated cutaneous, and mucocutaneous forms [1]. The species* Leishmania (Viannia) braziliensis*,* L. (V.) guyanensis*,* L. (L.) amazonensis,* and* L. (V.) panamensis* have been reported from northern Argentina [2–4], although the vast majority is caused by* L. (V.) braziliensis*. In this region,* Leishmania* spp. are transmitted by the bite of the female sandfly belonging to the genus* Lutzomyia* (Diptera, Psychodidae, and Phlebotominae).* Migonemyia migonei* (França 1920),* Evandromyia cortelezzii* (Brèthes 1923),* Evandromyia sallesi* (Galvão & Coutinho 1940),* Psathyromyia shannoni* (Dyar 1929),* Psathyromyia punctigeniculata* (Floch & Abonnenc, 1944), and* Nyssomyia neivai* (Pinto 1926) have been reported in northern Argentina [5, 6]; and particularly* Ny. neivai*,* Mg. migonei,* and* cortelezzii* complex have been proposed as potential vectors [7, 8].

The described scenarios of TL transmission in Argentina include four cycles' patterns: (a) wild cycle with transmission in primary or residual vegetation, (b) with eventual peridomestic transmission due to wild or secondary vegetation changes, (c) with peridomestic transmission in contiguous domiciles with the residual vegetation, and (d) peridomestic cycle in rural, ruralized periurban, or urban-rural interface environment [9]. However, the potential existence of urban transmission has been reported, which represents an important change in the transmission pattern paradigm of this disease at the regional scale [10]. Oran and San Martin departments (Salta province) are the areas with the greatest risk of transmission in the country, which reported the highest number of cases to the overall TL incidence in Argentina [10, 11].

In several areas of Latin America (including northern Argentina) the geographical distribution of TL overlaps with transmission areas of American trypanosomiasis (Chagas disease). The World Health Organization estimates that 8 to 10 million people are infected worldwide, mostly in Latin America where the disease is endemic [12]; this is caused by* Trypanosoma cruzi* and it is transmitted by several species of triatomine insects,* Triatoma infestans* being the most important in Argentina. In the last century, the progressive urbanization and the intensive migration of infected individuals increased the risk of transmission by blood transfusion and congenital route in nonendemic regions [13]. In restricted areas located in the east and northeast of Salta province, corresponding to the Gran Chaco ecoregion, vectorial transmission of* T. cruzi* still occurs but not in the rain forest ecoregion (Yunga ecoregion).

Mixed infections due to* Leishmania sp.* and* T cruzi* have been reported in patients showing clinical symptoms of TL, ranging between 12% and 70% [2, 14–16]. Prevalence of* Leishmania sp.* and* T. cruzi* mixed infection is unknown for northern Salta. Cross-reactivity between* T. cruzi* and* Leishmania* sp. infections has been reported when some serological tests were evaluated [2, 16, 17], possibly due to the close phylogenetic relationship between these parasites. The occurrence of* Leishmania* sp. and* T. cruzi* mixed infections also has therapeutic implications. Antimonial drugs used to treat leishmaniasis have potential cardiac toxicity [18–20], which is an important concern in patients infected by* T. cruzi* because about 30% of people infected by this parasite develop chronic cardiomyopathy [21].

In the present study, we examined the prevalence of single infections by* Leishmania* sp. and* T. cruzi* as well as the proportion of mixed infections due to both parasites in people living in the northern of Argentina. Demographic, behavioral, and environmental variables have also been studied as potential risk factors associated with transmission of cutaneous leishmaniasis. The epidemiologic pattern here observed can occur in several countries in Latin America and this research can provide information to optimize global and local prevention measures of public health.

## 2. Materials and Methods

### 2.1. Area and Population Studied

Hipólito Yrigoyen (Orán Department, Salta, Argentina) is located at 23°14′S, 64°16′W, 323 m.a.s.l. in large-scale farming and forest exploitation area, with subtropical climate. It is within the perimeter of the Pedemontana rain forest. This urban place has a population of 10 363 inhabitants (Primary Health Care Record, 2009). The study population included people who lived in Hipólito Yrigoyen between 2001 and 2011.

### 2.2. Study Design

This research included two cross-sectional studies, a tegumentary leishmaniasis case-control study, and the report of mixed infection for* Leishmania* sp. and* T. cruzi*.

A cross-sectional study was conducted in 2009 to determine the prevalence of* Leishmania* sp. (LP) taking into account the active cases of TL (ACTL) and total population of Hipólito Yrigoyen (HYTP: population censed by the PHC):

(1) $$\begin{array}{c} \text{LP}=\frac{\text{ACTL}}{\text{HYTP}}\times 100=\frac{\text{ACTL}}{10363}\times 100. \end{array}$$

**Figure 1:**
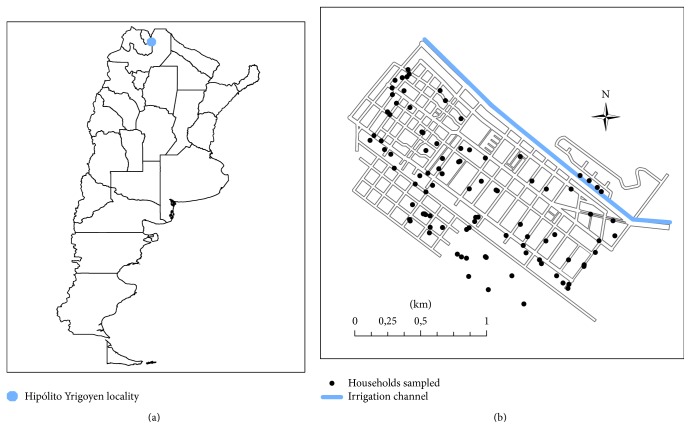
(a) HY within Argentina. (b) The black dots indicate households' distribution, where the sampling was conducted in the HY locality.

The seroprevalence of* T. cruzi* infection (TCSP) corresponding to 2009 was also calculated through a cross-sectional study. The sample size was 113 people. It was calculated considering an expected prevalence 5% with 4% of accuracy and confidence level of 95% using the Epidat software v3.1 (Epidat Xunta de Galicia, Santiago de Compostela, Spain and Pan-American Health Organization, Washington, DC). Seventy-nine quasi-randomly selected households were studied (Figure 1(b)). Of all household members, only those who wanted to participate voluntarily were selected (one person or more per household). The seroprevalence (TCSP) was calculated as the number of seropositive people for* T. cruzi* infection (TCP) over the sample size (SS):

(2) $$\begin{array}{c} \text{TCSP}=\frac{\text{TCP}}{\text{SS}}\times 100=\frac{\text{TCP}}{113}\times 100. \end{array}$$

**Figure 2:**
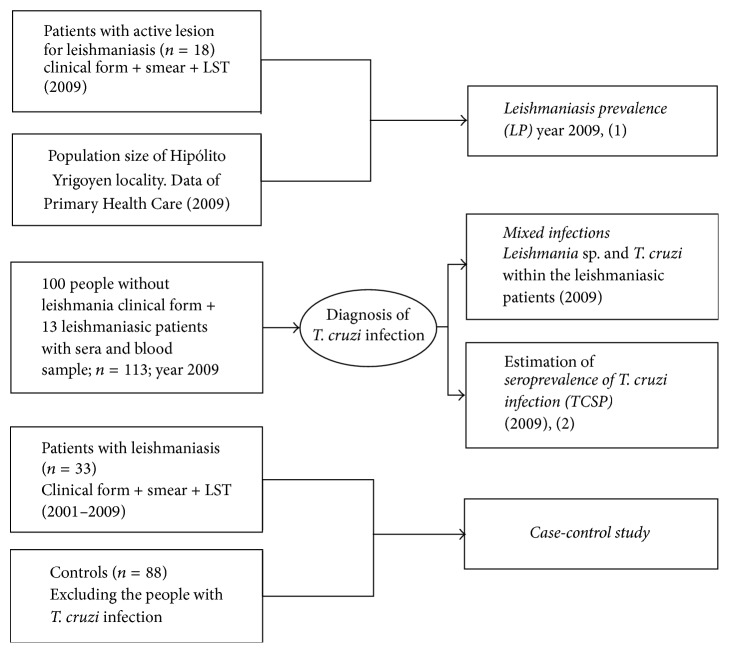
Flow chart showing the groups of patients and noninfected people included in prevalence (LP) and case-control study of leishmaniasis and* T. cruzi* seroprevalence (TCSP) infection. Mixed infections detected (*T. cruzi-Leishmania*) from 13 patients with active leishmaniasis ulcer in 2009 are also shown. Diagnostic of* T. cruzi* infection applied to the 100 people to detect both seropositive people to calculate* T. cruzi* seroprevalence and select the negative control people for the case-control study. People positive for ELISA-leishmaniasis or leishmanin skin test (LST) may have been exposed to the* Leishmania* parasite. The cross-reaction of both ELISA and leishmanin skin test with chagasic infection does not distinguish whether the person was exposed to* Leishmania* parasite or is infected with* T. cruzi*. Due to this, the people positive for Chagas lab tests were not included as controls in the case-control study.

Also, the mixed infection proportion within group of patients with TL in 2009 was calculated (Figure 2).

An unmatched case-control study was conducted to identify risk factors associated with TL cases. The TL cases included in the case-control study were patients living in Hipólito Yrigoyen and diagnosed between 2001 and 2009. The control population was selected within the cross-sectional sample used to calculate the* T. cruzi* seroprevalence infection (Figure 2). A survey was conducted in both cases and on the control people through a structured epidemiological questionnaire. After each person was interviewed, 6 mL of venous blood was drawn by clinical laboratory technicians and allowed to clot at room temperature. The sera were obtained by centrifugation at 3500 rotations per min for 5 min, then aliquoted into 1.5 mL tubes, and stored at −20°C until tested.

The variables registered were age, sex, occupation (exposure to vector bites at work), recreational habits (staying outdoors for more than 10 h and bathing in the river), personal preventive measures, household data (i.e., location, construction material, proximity to sites of possible development of sandflies, distance from crop fields and primary vegetation, knowledge about and application of preventive measures), living with people who were infected or had lesions, and aspects indicating knowledge about TL [22–24]. The data from the questionnaire were managed using the application EpiData Entry version 3.1 [25]; the resulting database was exported to the R statistical software for respective analysis.

The people included in each study group mentioned above were defined according to the diagnostic criteria described below.

### 2.3. Diagnostic Procedures

The patients were evaluated in the field by the personnel at Instituto de Investigaciones en Enfermedades Tropicales (IIET) at Universidad Nacional de Salta, the San Vicente de Paul Hospital in Orán, and Eva Peron Hospital in Hipólito Yrigoyen.

The sera and blood samples collected in the field were transported to the IIET for processing. The parasitological diagnostic of* Leishmania* was made in the IIET and the patients were derived from Hipólito Yrigoyen Hospital. Both diagnostic procedures such as serological, parasitological, and molecular techniques were performed following the protocols established in previous studies. The commercial kits were applied according to manufacturer's instructions.

### 2.4. Diagnostic Criteria

Diagnostic criteria included the following.

#### 2.4.1. Leishmaniasis Cases

Included were individuals who had lesions clinically compatible with TL and visualization of amastigotes of* Leishmania* sp. in Giemsa-stained smears and/or positive reaction of serum samples by enzyme-linked immunosorbent assay (ELISAg) homogenate protein of* L (V.) guyanensis* [15] and/or positive reaction to leishmanin skin test [4, 5].

#### 2.4.2. *T. cruzi* Infection

The subjects were considered infected with* T. cruzi* when serum samples by ELISA and Indirect hemagglutination (IHA) tests (Wiener Lab, Argentina) were reactive. The samples with discordant results between ELISA and IHA were examined by recombinant ELISA 3.0 (Wiener Lab, Argentina) and Immunofluorescence test [26] or Polymerase Chain Reaction (PCR) [27]. The recombinant ELISA 3.0 was reported as specific test for* T. cruzi* infection detection without cross-reaction with Leishmania [17, 28].

#### 2.4.3. Mixed Infections

Included were patients with TL and positive results for at least two tests for* T. cruzi* infection, mentioned above.

#### 2.4.4. Controls in Case-Control Study

Included were individuals living in Hipólito Yrigoyen who were not grouped as leishmaniasis cases and/or* T. cruzi* infected.

### 2.5. Data Analysis

The prevalence of* Leishmania* sp., seroprevalence of* T. cruzi* infections, and mixed infection proportion with 95% Confidence Intervals (CI) were calculated using the EPIDAT software version 3.1.

In the case-control study, the independent continuous and discreet variables were, respectively, categorized or dichotomized. Univariate and multivariate logistic regression (LR) analysis were carried out. The Odds Ratios (OR) and 95% CI were calculated to assess the link between the TL cases and potential risk factors. The variables with OR > 1 and *p* < 0.05 in univariate logistic regression analysis were tested in a multivariate analysis to establish a model involving the least number of variables that best explains the dependent variable (TL cases).

The final model was obtained using the stepwise technique, a procedure that combines forward method (it starts from a model only with the constant or independent variable, followed by progressive introduction of variables in the equation, provided they are significant) and backward method (all the variables are initially considered in the model, and those lacking significance are then progressively eliminated) [29]. The Akaike Information Criterion (AIC) was used as selection criterion. AIC calculation is based on minimization of the loss of information function, penalizing for the number of variables introduced that seeks the model that best adjusts to the data with the minimum number of possible variables, thus producing simpler models [30, 31]. The model that has been chosen minimizes the AIC. Data were considered statistically significant if *p* < 0.05. All statistical analysis for case-control study was performed using R software version 2.15.2 [32].

### 2.6. Ethical Approval

All the people included in the study agreed to participate by signing an informed consent form (ICF). The project and ICF were approved by the Ethics Committee of the School of Health Sciences at the National University of Salta and the “Fundación Huesped.”

## 3. Results

In 2009, only 18 cases of TL were diagnosed in Hipólito Yrigoyen, which represented a TL prevalence of 0.17% (CI 0.09–0.26). The age range of study patients was 7–69 years with an average of 35.45 ± 16.69 (SD).

Of the 113 samples analyzed to detect* T. cruzi* infection, 67 (59%) corresponded to females and 46 (41%) to males. Their age ranged between 7 and 74, with an average of 37.5 ± 17.3 (SD) years. There was no statistically significant difference in prevalence between males and females (*p* = 0.73). The seroprevalence for this infection was 9.73% (CI 3.83–15.64) in 2009. The frequencies of cases according to age and sex are summarized in Table 1.

Positive results were obtained for* T. cruzi* infection in three patients with TL in 2009 (Table 2), which represents a mixed infection proportion of 16.67% CI (3.58–41.42) within the group of 18 patients with TL.

The samples for the case-control study included 33 cases of TL (18 actives cases in 2009 and 15 cases registered in the 2001–2008 period) and 88 controls (Figure 2). The control group consisted of individuals without positive diagnosis for leishmaniasis and Chagas diseases according to criteria above described.

The variables that showed significant association (*p* < 0.05) with the presence of TL (*case*) in univariate LR analysis were* sex*,* age*, exposure to vector bites at work, staying outdoors for more than 10 h (*so*), bathing in the river, and living with people who were infected or had lesions during the study period (Table 3). These variables were included in a multivariate logistic regression analysis and final model obtained using the stepwise method. This model includes only 3 predictive variables (*sex*,* age,* and* so*) that explained the occurrence of TL cases (Table 3). The AIC value was 122.2

 The selected model: *CASE* = −2.82 + 1.80*∗SEX* + 2.04*∗AGE* + 1.42*∗SO*.

## 4. Discussion

In the north of Salta province, TL levels are hyperendemic in some sites and periods [33, 34]. This situation is worsened by the presence of cases of Chagas disease, which may further generate cases of mixed infections, causing a synergistic problem for the health care system. In the locality Hipólito Yrigoyen, TL prevalence values of 0.17, 0.79, and 0.18% were previously reported [33, 34]. The prevalence calculated in the present study (0.17%; CI 0.08–0.26) is similar, which indicates a level of active transmission that persists over time. However, high incidence foci are likely to occur in short exposure periods in this area [5].

In the case-control study, the associated variables would be reflecting the existence of a complex pattern of transmission. Male sex and staying outdoors for more than 10 hours would be indicators of a sylvatic mode of transmission, facilitated by labour, subsistence, or recreational activities (in rural environments and/or deforestation areas, or hunting and fishing activities), as it is indicated in regions where TL is endemic [9, 24, 33]. The significantly higher proportion of infected children compared to that of adults found in this analysis (OR = 7.73; CI: 2.05–29.16) suggests the existence of other patterns of transmission in Hipólito Yrigoyen. The incidence of TL in children has been cited as an indicator of peridomiciliary transmission, especially in localities adjacent to primary and/or secondary vegetation [4, 22–24, 33].

In addition, a high density of sandflies has been detected in the vegetation near to the irrigation channels located in the outskirts of the city (Figure 1) [35], showing a species diversity similar to that found in a nearby place where there was a high rate of infection [5, 36]. Many families go to these sites for recreational purposes in times of high temperature and risk leishmaniasis transmission (7 pm to 10 pm approximately) with the consequent risk of being bitten by infected sandflies and contracting leishmaniasis, as reported in a study of spatial distribution of TL cases [37].

The presence of active TL cases among elderly people that remain mostly in their houses and of sandflies in the center of the town [35] offers another plausible epidemiologic situation of disease transmission (but with low probability) in urban environments because lower abundance of sandflies was recorded here [35]. Indeed, in Hipólito Yrigoyen, house courtyards have vegetation patches that can be colonized by sandflies from the periphery, according to the characteristics of metapopulation dynamics [10].

On the other hand, in the study area, the possibility of vector-borne transmission of* T. cruzi* has been discarded, because no insects or indicators of their presence have been found in the annual activities of entomological surveillance of triatomines carried out by Primary Health Care System in recent and historical monitoring. Thus, cases of* T. cruzi* infection in Hipólito Yrigoyen would be associated with migratory processes (movement principally of rural populations from Argentina and Bolivia of the Gran Chaco ecoregion where Chagas disease is endemic) [13]. The* T. cruzi* seroprevalence found in this work is low compared with the prevalence value observed in rural populations of endemic areas (25%) [38], and it is high compared with other regions without endemic transmission [39]. In turn, infected children may have acquired infection by congenital transmission, as reported in previous studies of this type of transmission in the province of Salta [40].

The proportion of* T. cruzi-Leishmania* sp. mixed infection within the group of patients with TL reported in the north of Salta reaches 30 and 40% [2, 14, 15] and does not show differences from the percentage obtained in this study. Knowing the level of this condition in the population allows us to explore the factors involved in the origin and persistence of mixed infections. In addition, because antimonials are cardiotoxic, a careful diagnosis and implementation of alternative treatments are needed to avoid further complications.

The complex situation in Hipólito Yrigoyen in reference to TL is aggravated by the coexistence of* T. cruzi* infection. The transmission pattern involves mainly natural areas, but the possibility of a peridomiciliary transmission in the outskirts of the city cannot be ruled out. This situation demands the involvement of different stakeholders to control the magnitude of disease incidence, implementing prevention strategies and taking into account biogeographical and sociocultural characteristics, as well as human-induced environmental changes and situations that pose a risk.

The present work provides epidemiological information of potential determinants of TL occurrence in Hipólito Yrigoyen, its magnitude, and situation of* T. cruzi* infection in the same area. This information is useful for the local health system because it may contribute to a better planning of the surveillance systems and to the design of prevention strategies in the area. These epidemiological patterns of mixed infections can occur in other countries were* T. cruzi* transmission does not exist or was interrupted and tegumentary leishmaniasis is endemic.

## Figures and Tables

**Table 1:** Number of individuals included in the study, percentage (%) of individuals infected with *T. cruzi* according to sex and age group. ^a^Indication of the overall prevalence in Hipólito Yrigoyen for 2009. *i*: infected individuals according to sex and age; *n*: total sample in seroprevalence of *T. cruzi *study.

Sex	Female *i*/*n* (%)	Male *i*/*n* (%)
Total infected	5/67 (7)	6/46 (13)

Age	*n*	Infected (%)

10–35	62	4 (6.45)
36–55	28	3 (10.7)
>55	23	4 (10.4)

Total	**113**	**11 (9.73)** ^a^

**Table 2:** Results of samples with mixed infection. SMEAR: visualization of amastigotes of *Leishmania* sp. in Giemsa-stained smears; ELISAg: ELISA based on homogenate protein of *L (V.) guyanensis*; LST: reaction to leishmanin skin test; ELISA Rec: recombinant ELISA Kit; IHA: indirect hemagglutination; TIF: test of Immunofluorescence; PCR: Polymerase Chain Reaction. nd: no data (samples were not evaluated with TIF).

		*Leishmania* infection tests			*T. cruzi* infection tests			
		Smear	ELISAg	LST	ELISA Rec.	IHA	TIF	PCR
Patient code	HI 54	+	+	+	+	+	+	−
	HI 55	+	+	+	−	+	nd	+
	HI 58	+	+	+	+	+	nd	+

**Table 3:** Crude OR and adjusted OR for the factors associated with the presence of tegumentary leishmaniasis. OR, Odds Ratio; CI, Confidence Interval. ^a^Significant association at *p* < 0.05; ^b^highly significant association at *p* < 0.01; ^c^highly significant association at *p* < 0.001.

Logistic regression	OR (CI 95%) univariate analysis	OR (CI 95%) multivariate analysis
*Sex *		
Female	1	1
Male	5.47 (2.21–13.54)^a^	6.05 (2.2–16.64)^c^
*Age (years)*		
>16	1	1
7–15	4.37 (1.39–13.8)^b^	7.73 (2.05–29.16)^b^
*Exposure to vector bites at work*		
Unexposed	1	
Exposed	2.68 (1.14–6.3)^a^	
*Permanence > 10 hours outdoors (SO)*		
No	1	1
Yes	4.67 (1.23–17.78)^a^	4.16 (0.97–17.77)^a^
*Bathing in the river*		
No	1	
Yes	3.89 (1.59–9.54)^a^	
*Living with infected people*		
No	1	
Yes	3.17 (1.23–8.15)^a^	

## References

[1] Krolewiecki A. J., Romero H. D., Cajal S. P. (2007). A randomized clinical trial comparing oral azithromycin and meglumine antimoniate for the treatment of American cutaneous leishmaniasis caused by *Leishmania (Viannia) braziliensis*. *The American Journal of Tropical Medicine and Hygiene*.

[2] Frank F. M., Fernández M. M., Taranto N. J. (2003). Characterization of human infection by *Leishmania* spp. in the Northwest of Argentina: immune response, double infection with *Trypanosoma cruzi* and species of *Leishmania* involveds. *Parasitology*.

[3] Marco J. D., Barroso P. A., Mimori T. (2012). Polymorphism-specific PCR enhances the diagnostic performance of American tegumentary leishmaniasis and allows the rapid identification of *Leishmania* species from Argentina. *BMC Infectious Diseases*.

[4] Locatelli F. M., Cajal S. P., Barroso P. A. (2014). The isolation and molecular characterization of *Leishmania* spp. from patients with American tegumentary leishmaniasis in northwest Argentina. *Acta Tropica*.

[5] Krolewiecki A. J., Gil J. F., Quipildor M. (2013). Restricted outbreak of American tegumentary leishmaniasis with high microfocal transmission. *American Journal of Tropical Medicine and Hygiene*.

[6] Salomón O. D., Wilson M. L., Munstermann L. E., Travi B. L. (2004). Spatial and temporal patterns of phlebotomine sand flies (Diptera: Psychodidae) in a cutaneous leishmaniasis focus in northern Argentina. *Journal of Medical Entomology*.

[7] de Pita-Pereira D., Alves C. R., Souza M. B. (2005). Identification of naturally infected *Lutzomyia intermedia* and *Lutzomyia migonei* with *Leishmania (Viannia) braziliensis* in Rio de Janeiro (Brazil) revealed by a PCR multiplex non-isotopic hybridisation assay. *Transactions of the Royal Society of Tropical Medicine and Hygiene*.

[8] Córdoba-Lanús E., De Grosso M. L., Piñero J. E., Valladares B., Salomón O. D. (2006). Natural infection of *Lutzomyia neivai* with *Leishmania* spp. in northwestern Argentina. *Acta Tropica*.

[9] Salomón O. D., Orellano P. W., Quintana M. G. (2006). Transmission of tegumentary leishmaniasis in Argentina. *Medicina*.

[10] Gil J. F., Nasser J. R., Cajal S. P. (2010). Urban transmission of american cutaneous leishmaniasis in Argentina: spatial analysis study. *The American Journal of Tropical Medicine and Hygiene*.

[11] Salomon O. D., Rosa J. R., Stein M. (2008). Phlebotominae (Diptera: Psycodidae) fauna in the Chaco region and Cutaneous Leishmaniasis transmission patterns in Argentina. *Memórias do Instituto Oswaldo Cruz*.

[12] World Health Organization (2013). Research priorities for chagas disease, human african trypanosomiasis and leishmaniasis. *WHO Technical Report Series*.

[13] Dias J. C. P. (2013). Human chagas disease and migration in the context of globalization: some particular aspects. *Journal of Tropical Medicine*.

[14] Chiaramonte M. G., Frank F. M., Furer G. M., Taranto N. J., Margni R. A., Malchiodi E. L. (1999). Polymerase chain reaction reveals *Trypanosoma cruzi* infection suspected by serology in cutaneous and mucocutaneous leishmaniasis patients. *Acta Tropica*.

[15] Gil J. F., Hoyos C. L., Cimino R. O. (2011). Role of three ELISA tests using promastigote homogenates of *Leishmania braziliensis, L. amazonensis* and *L. guyanensis* in the diagnosis of tegumentary leishmaniasis. *Medicina*.

[16] Vega Benedetti A. F., Cimino R. O., Cajal P. S. (2013). Performance of different *Trypanosoma cruzi* antigens in the diagnosis of Chagas disease in patients with American cutaneous leishmaniasis from a co-endemic region in Argentina. *Tropical Medicine & International Health*.

[17] Gil J. F., Cimino R. O., Lopez Quiroga I. (2011). Reactivity of GST-SAPA antigen of *Trypanosoma cruzi* against sera from patients with Chagas disease and leishmaniasis. *Medicina*.

[18] Lawn S. D., Armstrong M., Chilton D., Whitty C. J. M. (2006). Electrocardiographic and biochemical adverse effects of sodium stibogluconate during treatment of cutaneous and mucosal leishmaniasis among returned travellers. *Transactions of the Royal Society of Tropical Medicine and Hygiene*.

[19] Oliveira L. F., Schubach A. O., Martins M. M. (2011). Systematic review of the adverse effects of cutaneous leishmaniasis treatment in the New World. *Acta Tropica*.

[20] Gamboa Latorre L. F., Becerra Mateus J. A. (2012). Cardiotoxicidad por antimoniales. *Revista Salud Bosque*.

[21] Malik L. H., Singh G. D., Amsterdam E. A. (2015). The epidemiology, clinical manifestations, and management of chagas heart disease. *Clinical Cardiology*.

[22] Sosa-Estani S., Segura E. L., Gomez A. (2001). Cutaneous leishmaniasis in Northern Argentina: identification of risk factors in a case-cohort study of three municipalities in Salta. *Revista da Sociedade Brasileira de Medicina Tropical*.

[23] Yadon Z. E., Rodrigues L. C., Davies C. R., Quigley M. A. (2003). Indoor and peridomestic transmission of American cutaneous leishmaniasis in northwestern Argentina: a retrospective case-control study. *American Journal of Tropical Medicine and Hygiene*.

[24] Pedrosa F. D. A., Ximenes R. A. D. A. (2009). Sociodemographic and environmental risk factors for American cutaneous leishmaniasis (ACL) in the State of Alagoas, Brazil. *American Journal of Tropical Medicine and Hygiene*.

[25] Lauritsen J. M., Bruus M. (2005). *EpiData Entry, Version 3.1. A Comprehensive Tool for Validated Entry and Documentation of Data*.

[26] Alvarez M., Cerisola J. A., Rohwedder R. W. (1968). Immunofluorescence test in the diagnosis of Chagas' diseases. *Boletín Chileno de Parasitología*.

[27] Britto C., Cardoso M. A., Monteiro Vanni C. M. (1995). Polymerase chain reaction detection of *Trypanosoma cruzi* in human blood samples as a tool for diagnosis and treatment evaluation. *Parasitology*.

[28] Caballero Z. C., Sousa O. E., Marques W. P., Saez-Alquezar A., Umezawa E. S. (2007). Evaluation of serological tests to identify *Trypanosoma cruzi* infection in humans and determine cross-reactivity with *Trypanosoma rangeli* and *Leishmania* spp.. *Clinical and Vaccine Immunology*.

[29] Domínguez-Almendros S., Benítez-Parejo N., Gonzalez-Ramirez A. R. (2011). Logistic regression models. *Allergologia et Immunopathologia*.

[30] Chongsuvivatwong V. (2008). *Analysis of Epidemiological Data Using R and Epicalc*.

[31] Rodríguez del Águila M. M., Benítez-Parejo N. (2011). Simple linear and multivariate regression models. *Allergologia et Immunopathologia*.

[32] R Core Team R: A language and environment for statistical computing. http://www.R-project.org/.

[33] Sosa-Estani S., Segura E. L., Salomón O. D. (2000). Tegumentary leishmaniasis in Northern Argentina: distribution of infection and disease, in three municipalities of Salta, 1990–1992. *Revista da Sociedade Brasileira de Medicina Tropical*.

[34] Salomón O. D., Estani S. S., Canini L., Lanus E. C. (2001). Tegumentary leishmaniasis in an area with epidemic levels of transmission, Salta, Argentina, 1998. *Medicina B: Aires*.

[35] Chanampa M. M. (2012). *Monitoring sandflies of Lutzomyia genus and associated environmental variables, in the town of Hipólito Yrigoyen [M.S. Biologist thesis]*.

[36] Quintana M. G., Salomon O. D., De Grosso M. S. (2010). Distribution of phlebotomine sand flies (Diptera: Psychodidae) in a primary forest-crop interface, Salta, Argentina. *Journal of Medical Entomology*.

[37] Rosales J. C., Yang H. M., Ibarra A., Barraza R. (2008). Análise da distribuição espacial dos casos de leishmaniose tegumentar americana em Hipólito Yrigoyen, Orán, Salta, Argentina, no período 2005-2006. *Boletim Epidemiológico Paulista*.

[38] Diosque P., Padilla A. M., Cimino R. O. (2004). Chagas disease in rural areas of Chaco Province, Argentina: epidemiologic survey in humans, reservoirs, and vectors. *The American Journal of Tropical Medicine and Hygiene*.

[39] Klein N., Hurwitz I., Durvasula R. (2012). Globalization of Chagas disease: a growing concern in nonendemic countries. *Epidemiology Research International*.

[40] Negrette O. S., Mora M. C., Basombrío M. Á. (2005). High prevalence of congenital *Trypanosoma cruzi* infection and family clustering in Salta, Argentina. *Pediatrics*.

